# Quantitative Detection Technology for Geometric Deformation of Pipelines Based on LiDAR

**DOI:** 10.3390/s23249761

**Published:** 2023-12-11

**Authors:** Min Zhao, Zehao Fang, Ning Ding, Nan Li, Tengfei Su, Huihuan Qian

**Affiliations:** 1 Shenzhen Institute of Artificial Intelligence and Robotics for Society, Shenzhen 518129, China; 2 School of Science and Engineering, The Chinese University of Hong Kong, Shenzhen 518172, China; 3 Institute of Robotics and the Intelligent Manufacturing, Shenzhen 518172, China; 4 Shenzhen Water SCI&Tech. Development Co., Ltd., Shenzhen 518035, China

**Keywords:** LiDAR, point cloud, pipeline deformation, quantitative detection

## Abstract

This paper introduces a novel method for enhancing underground pipeline inspection, specifically addressing limitations associated with traditional closed-circuit television (CCTV) systems. These systems, commonly used for capturing visual data of sewer system deformations, heavily rely on subjective human expertise, leading to limited accuracy in detection. Furthermore, their inability to perform quantitative analyses of deformation extent hampers overall inspection effectiveness. Our proposed method leverages laser point cloud data and employs a 3D scanner for objective detection of geometric deformations in underground pipe corridors. By utilizing this approach, we enable a quantitative assessment of blockage levels, offering a significant improvement over traditional CCTV-based methods. The key advantages of our method lie in its objectivity and quantification capabilities, ultimately enhancing detection reliability, accuracy, and overall inspection efficiency.

## 1. Introduction

Urban underground pipelines serve as vital components of cities, facilitating the transportation of crucial resources such as water and natural gas. They play a pivotal role in supporting daily life and industrial production, functioning as the lifelines of cities and contributing significantly to smart city development. However, the presence of deformations in these pipelines can pose safety hazards, leading to collapses and economic losses. These pipelines are typically concealed from view, which allows internal deformations to accumulate over time. Consequently, it is widely acknowledged that conducting regular inspections during both the construction and operational phases of underground pipelines is crucial for maintaining and repairing existing and potential deformations. These inspections are essential for ensuring the reliability of underground pipelines and mitigating risks by identifying and addressing issues in a timely manner. Underground pipelines are susceptible to damage from various factors, including working conditions and the surrounding environment. However, the intricate nature of pipeline infrastructure poses challenges in locating and detecting common deformations. Traditional pipeline inspections have heavily relied on manual labor, which is both labor-intensive and hazardous. Recent technological advancements have introduced robots as alternatives to human workers, which can enter confined spaces and capture videos to avoid potential risks.

Existing pipeline inspection techniques can generally be categorized into visual inspection and sensor-based detection [1]. Visual inspection involves manual maintenance conducted by personnel who directly inspect the pipeline using techniques like mirrors and diving. However, direct visual inspection comes with disadvantages such as high workload, low accuracy, and the potential for missed defects due to its reliance on human labor. Moreover, ensuring the safety of inspection personnel can be challenging and is dependent on the pipeline’s size and working environment. In contrast, sensor-based detection methods utilize specialized equipment designed for specific pipeline conditions and sizes, reducing the dependence on human resources and mitigating potential risks. Sensor-based pipeline inspection platforms primarily consist of robots equipped with specialized sensor devices that enter the pipeline to perform inspections and gather information about its operational conditions. Depending on the type of sensor equipment used, various detection technologies are available, including infrared thermography systems [2], ground-penetrating radar systems [3], ultrasound detection systems [4], and magnetic leakage detection technology [5]. However, these detection methods often focus on specific defect types and may lack universal applicability.

With the rapid advancement of imaging sensors and improvements in machine computing capabilities, Closed-Circuit Television (CCTV) inspection [6] has become a mainstream technology for internal pipeline inspections. This technique involves deploying robots equipped with cameras into the pipeline to perform video-based inspections [7]. The captured images are uploaded to a central unit, where they are examined and interpreted by trained engineers. Compared with sensor-based detection methods, CCTV inspection offers advantages like lower cost and simplified operation. However, as the total length of urban underground pipeline networks continues to increase, inspection methods that rely on manually assessing large amounts of video footage become time-consuming and inefficient. This inefficiency leads to inadequate inspection and increased risk of human error. As a result, current research on CCTV technology focuses on transitioning from human-dependent pipeline condition assessment to automated machine recognition [8,9]. For instance, Dang et al. [10] proposed a multi-framework ensemble detection model that extracts precise defect information from key frames of CCTV videos by leveraging different frameworks’ strengths. Cheng and Wang [11] used the Faster R-CNN technique to identify defects in sewage pipelines and investigated the impact of network hyper-parameters on recognition accuracy. Jiao et al. [12] introduced a steerable autoencoder-based framework for anomaly detection in CCTV frames. Yin et al. [13] applied the YOLOv3 network with a simulated annealing algorithm to achieve real-time defect detection in sewage pipelines. Ma et al. [14] proposed a real-time processing and segmentation framework for automatic deblurring of pipeline defect images using a GAN network.

Despite progress in these recognition techniques, the limitations of two-dimensional imaging technology still hinder accurate assessments of crucial parameters such as changes in pipeline wall thickness and deformation severity, limiting comprehensive pipeline structure analysis. To address these challenges, the adoption of three-dimensional (3D) laser scanning emerges as a more viable solution. This technology utilizes laser scanners for conducting internal pipeline scans, capturing point cloud data quickly and precisely. This supports the accurate detection and reconstruction of internal pipeline conditions, including cracks, corrosion, and deformations in confined spaces. Researchers have proposed various methods to enhance the application of laser point cloud imaging technology in pipeline inspection. Cheng et al. [15] introduced a prior-based pipeline reconstruction method using convolutional networks to detect pipeline components and fit them into the original point cloud data. In the work by Meng et al. [16], hybrid object detection and tracking for cooperative perception are achieved using 3D LiDAR. Pang et al. [17] utilized laser radar for point cloud segmentation and reconstruction, achieving reconstruction and error assessment specifically targeted at defects. Nevertheless, prevailing methods exhibit a limitation in the capability to intuitively conduct quantitative analysis of internal geometric deformations.

In response to this limitation, this paper proposes a method for the quantitative detection of internal spatial deformations within subterranean pipelines, leveraging laser point cloud data. This approach integrates traditional pipeline robots with high-precision radar, thereby enhancing the accuracy and precision of deformation assessment. Furthermore, the incorporation of a grid-based algorithm serves to delineate the contours of the inner pipeline walls, which facilitates the determination of the section’s loss area. Following experimental validation, this methodology proves instrumental in enabling the detection and precise assessment of pipeline damage.

The remainder of this paper is organized as follows. Section 2 introduces the hardware structural design of the overall experimental system. In Section 3, the framework of the proposed method for internal pipeline wall scans based on high-density point cloud data is described. In Section 4, the detection rate and detection accuracy of the proposed method are analyzed for pipelines with different levels of blockage. Finally, Section 5 concludes the paper and discusses future work.

## 2. Hardware Design

**Figure 1:**
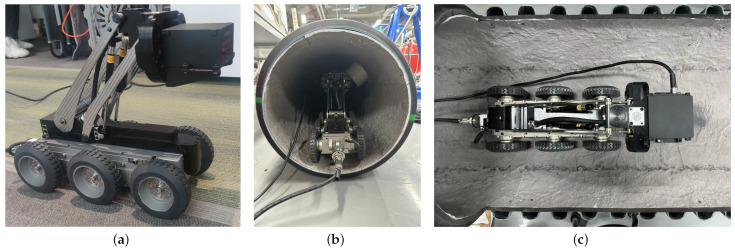
Three-dimensional scanning system platform for pipelines (**a**) Diagram of the execution mechanism (**b**) Side view of field inspection (**c**) Top view of field inspection.

The detection system comprises two main components: the execution mechanism and the sensing and detection mechanism. The execution mechanism is represented by a four-wheel-drive smart car, as illustrated in Figure 1a. The sensing and detection module, integrated into the mobile platform, comprises an Inertial Measurement Unit (IMU) module and a Light Detection and Ranging (LiDAR) module. The IMU module is incorporated into the system to precisely measure and track the pose of the LiDAR. The LiDAR system employs the LIVOX HAP, leveraging high-speed non-repetitive scanning technology and multi-line packaged lasers.

**Figure 2:**
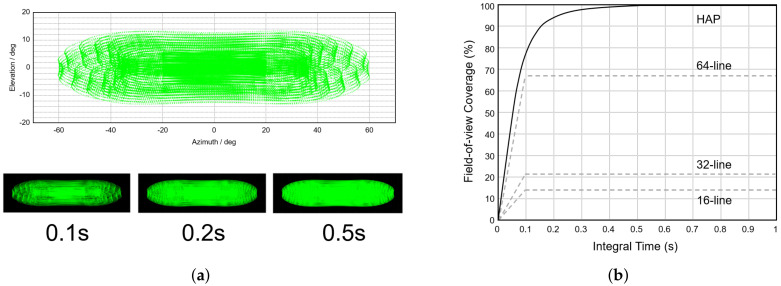
Scan point cloud distribution (**a**) Typical scan point cloud distribution and comparison of scan point cloud distributions with different integration times (**b**) Comparison of integration time and field of view coverage of HAP and different LiDARs.

Figure 2a displays the typical point cloud distribution at 0.1s integration time and the field-of-view coverage of the HAP at different integration times. In this representation, the central area exhibits a higher point density, with an average scanning interval of approximately 0.2 degrees, while the point density decreases towards the ends, with an average scanning interval of approximately 0.3 degrees. Additionally, it is noteworthy that a longer integration time has a subtle effect on the field-of-view range but enhances the coverage rate. This enhancement allows for capturing finer details within the field of view. In Figure 2b, the field-of-view coverage of the HAP is compared with 16-line, 32-line, and 64-line LiDARs at different integration times. Remarkably, the HAP achieves a field-of-view coverage of approximately 80% when the integration time is less than 0.1 s, surpassing that of a typical 64-line mechanically rotating LiDAR. As the integration time increases, more details are captured, and the point cloud coverage rate improves to over 99%. These results underscore the outstanding coverage and resolution capabilities of the HAP, making it highly suitable for 3D scanning and modeling in underground pipeline scenarios. However, it is also crucial to acknowledge limitations, particularly concerning smooth surfaces, such as potential challenges in scanning the internal surface contours clearly when residual water is present at the bottom of the pipeline. This limitation arises due to potential interference caused by water residue, impacting the clarity of the internal surface profile scans.

## 3. Methodologies

**Figure 3:**
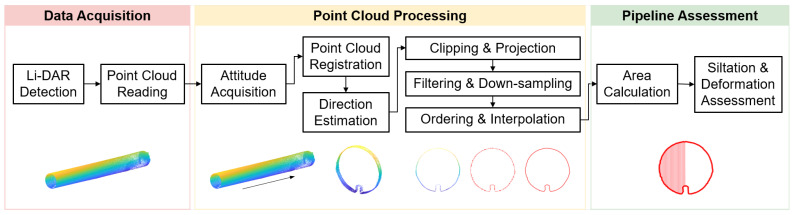
Point cloud processing workflow.

The laser scanning system enables the acquisition of a large volume of high-precision 3D point cloud data from various perspectives, facilitating the efficient reconstruction of the 3D pipeline network model. The workflow for quantitative detection of internal deformations in pipelines based on laser scanning, as illustrated in Figure 3, involves the following key steps.

First, real-time or recorded point cloud information is obtained through point cloud reading. Multiple point clouds from various time frames are then registered and combined to form a comprehensive dataset representing the internal space of the pipeline. Based on the variations in the coordinate information of the point cloud, the direction of the pipeline is determined. Using this information, the point cloud is cropped and segmented along the pipeline direction at fixed intervals. Subsequently, the cropped 3D point cloud information is projected onto a 2D cross-section. Through the application of filtering and downsampling techniques, the contour information of the local cross-section’s point cloud is extracted. Once the contour point cloud information is obtained, it undergoes further processing to generate an ordered point cloud, and interpolation techniques are applied to enhance the spatial distribution density of the point cloud. Finally, the interpolated pipeline contour is used to calculate the area of the detected deformation. This process provides specific numerical information for deformation evaluation and offers robust data support for quantitative deformation detection.

### 3.1. Point Cloud Data Acquisition

Point cloud data is a dataset that contains rich information in the Cartesian coordinate system, including three-dimensional coordinates (X, Y, Z), color, classification values, intensity values, and timestamps. By scanning the internal structure of the pipeline, spatial point cloud data from different positions within the pipeline can be obtained, forming the foundation for subsequent analysis. To assess the effectiveness and accuracy of the proposed method, an experimental pipeline blockage model is created. This model involves placing obstructions of varying volumes inside the pipeline to simulate different blockage scenarios. This setup allows for the evaluation of the proposed method’s performance in detecting and quantifying internal deformation extent under varying blockage conditions. The intelligent vehicle, equipped with laser-based 3D scanning capabilities, is then deployed into the pipeline to capture the point cloud information. To enhance the accuracy of internal reconstruction, point cloud data from different time frames are overlapped and combined to achieve more precise internal reconstruction results.

### 3.2. Point Cloud Preprocessing

The point cloud generated from laser scanning typically contains a substantial volume of data and is often accompanied by noise, especially when scanning complex objects like pipelines. Additionally, the complexity of the pipeline environment can result in missing data points, posing challenges for the reconstruction of the pipeline network model. To achieve accurate reconstruction of the pipeline network model, preprocessing operations are applied to the point cloud data. These preprocessing steps include point cloud filtering, registration, segmentation, and projection. Filtering techniques are employed to remove noise and outliers from the point cloud data, thereby enhancing the quality and reliability of the reconstructed model. Point cloud registration involves aligning multiple point clouds acquired from different viewpoints or time frames to create a unified and complete representation of the pipeline’s internal structure. Point cloud segmentation and projection entail dividing the point cloud into meaningful regions based on geometric properties or clustering algorithms. This process enables further analysis and processing of specific pipeline components. Projection techniques are then applied to project the segmented point cloud onto a two-dimensional representation, facilitating subsequent analysis and visualization. These preprocessing steps optimize the point cloud data while preserving the geometric characteristics of the pipeline, leading to a more detailed and comprehensive representation of the pipeline network.

#### 3.2.1. Point Cloud Denoising

During the process of laser scanning, the received point cloud signals can be influenced by various factors, such as the surface characteristics of the pipeline inner wall, scanning environment, and system limitations. Consequently, the acquired point cloud data often contain noise and outliers, necessitating denoising operations to enhance the effectiveness of subsequent point cloud processing. Point cloud denoising typically involves several methods, including outlier removal, normal-based filtering, and voxel-based filtering. These denoising techniques effectively eliminate or smooth out the noise and outliers present in the point cloud, thereby improving the accuracy and effectiveness of subsequent point cloud processing.

#### 3.2.2. Point Cloud Registration

Point cloud registration is the process of aligning multiple point clouds to achieve a more accurate 3D model in a common coordinate system. Various methods can be employed for point cloud registration, including target-based registration, feature-based registration, measurement-based registration, and hybrid registration. Among these methods, the Iterative Closest Point (ICP) algorithm is a widely used automatic point cloud registration method. The ICP algorithm aims to find correspondences between points in the source and target point clouds and iteratively optimizes the rotation and translation parameters using the least squares method. In this work, the ICP algorithm is primarily used for point cloud registration, following these principles:

Assuming the presence of two sets of unregistered point cloud data, denoted as $X=x_{1},x_{2},\ldots ,x_{n}$ and $Y=y_{1},y_{2},\ldots ,y_{n}$. Here *X* represents the source point cloud, *Y* represents the target point cloud, and $y_{i}$ denotes the point in the target point cloud *Y* that is closest to $x_{i}$ in the source point cloud. The objective is to find an optimal rigid transformation matrix, typically represented by an affine transformation matrix RT, which can accurately align the source point cloud *X* with the target point cloud *Y*. This matrix satisfies the equation:

(1) $$\forall i,\;x_{i}=Ry_{i}+t$$

Here, *R* represents the rotation matrix and *t* represents the translation matrix. The algorithm utilizes the least squares method to quantify the closeness between the two point clouds and defines an error function as follows:

(2) $$E\left(R,t\right)=\frac{1}{2}\sum_{i=1}^{n}{\left\|x_{i}-\left(Ry_{i}+t\right)\right\|}^{2}$$

The ICP problem, as expressed in Equation (2), is reformulated to seek the optimal rotation matrix *R* and translation matrix *t* that minimize the error function E(R,t). The solution approach entails resolving for the optimal *R* and *t* by tackling the optimization problem in relation to deviations of $x_{i}$ and $y_{i}$ from the mean.

The demeaned point clouds sets as $X^{\prime }$ and $Y^{\prime }$. The optimal rotation matrix *R* can now be found by minimizing the error function:

(3) $$R^{*}=\arg\min E\left(R\right)=\arg\min_{R}\frac{1}{2}\sum_{i=1}^{n}{\left\|x_{i}^{\prime }-Ry_{i}^{\prime }\right\|}^{2}$$

To compute $R^{*}$, the algorithm defines the matrix $W=\sum_{i=1}^{n}y_{i}^{\prime }x_{i}^{\prime T}$, which is a 3×3 matrix. Performing the Singular Value Decomposition (SVD) of *W* yields:

(4) $$W=U\Sigma V^{T}$$

When *W* is full rank, there exists a unique combination of *U* and *V*, which allows us to derive the optimal rotation matrix *R* and the translation vector *t*:

(5) $$R^{*}=UV^{T}$$

(6) $$t=x_{i}^{\prime }-R*y_{i}^{\prime }$$

**Figure 4:**
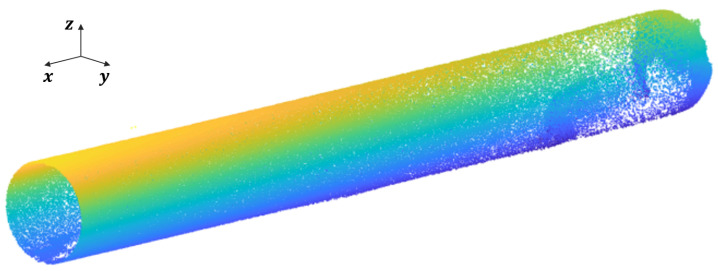
Point cloud scan diagram inside the pipeline.

Hence, the optimal solution for (R,t) is obtained. Using this approach, all 34 remaining point cloud sets are registered. The resulting registered point cloud data is depicted in Figure 4.

#### 3.2.3. Point Cloud Segmentation and Projection

The recognition of the pipeline’s orientation is primarily achieved by comparing the coordinate values of the point cloud in the X, Y, and Z directions. Since the pipeline has a cylindrical structure, the variation range of the point cloud data in the Y and Z coordinates is relatively small. Based on the coordinate system of the laser scanner, the main orientation of pipelines can be determined by analyzing the coordinate values along the X-axis. Therefore, the clipping operation on the point cloud data is mainly performed along the X-axis.

**Figure 5:**
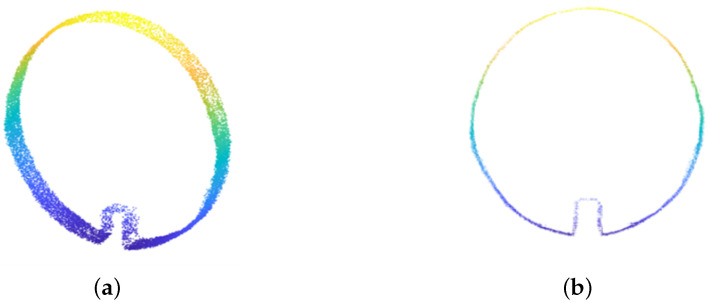
Clipped segment and projection of typical pipeline sections (**a**) Point cloud segment (**b**) Projection of segment.

Taking into account the structural characteristics of the pipeline and the limitations of computational resources, the dataset is clipped at intervals of 0.1 m along the X-axis. The clipped point cloud is then projected onto the YZ-plane, forming a dense distribution of 2D point clouds, as shown in Figure 5. To reduce the data size and improve computational efficiency and modeling speed without compromising important geometric features, downsampling is applied to the projected point cloud. Common point cloud downsampling algorithms include spatial segmentation-based methods and curvature-based methods. In this experiment, the point cloud is downsampled with an interval of 20 mm.

### 3.3. Internal Profile Extraction and Quantitative Detection

Based on the point cloud preprocessing discussed in Section 3.2, it is essential to evaluate the blockage level information of the pipeline cross-sectional contour point cloud. Various methods have been proposed for point cloud boundary extraction, including latitude-longitude scanning, grid partitioning, normal estimation, and an alpha shapes algorithm. Considering the specific characteristics of each algorithm, the grid partitioning method is adopted in this experiment to detect the boundary of the pipeline point cloud. Specifically, the grid partitioning method includes the following three steps.

#### 3.3.1. Grid Partitioning and Hollow Cell Filling

The widely used grid partitioning method is the uniform gridding approach. First, the acquired 3D data point cloud set $q=q_{1},q_{2},\ldots ,q_{n}$ is mapped to a 2D plane by applying coordinate transformation, forming a point cloud set $p=p_{1},p_{2},\ldots ,p_{n}$ within the plane. Here, $q_{i}={\left(x_{i},y_{i},z_{i}\right)}^{T}$ and $p_{i}={\left(x_{i},y_{i}\right)}^{T}$. After obtaining the mathematical representation of the point set, all points are traversed to determine the maximum and minimum values of *x* and *y*, denoted as $x_{\max}$, $y_{\max}$, $x_{\min}$ and $y_{\min}$, respectively. This establishes the minimum bounding box for the point set. Based on this, the grid size *L* can be calculated as:

(7) $$L=\sqrt{\frac{\left(x_{max}-x_{min}\right)\left(y_{max}-y_{min}\right)}{n}}$$

Correspondingly, the number of grids in the *x* and *y* directions, denoted as $x_{num}$ and $y_{num}$, can be calculated as:

(8) $$\left\{\begin{array}{cc} x_{num} & =\left\lfloor \frac{x_{max}-x_{min}}{L}\right\rfloor +1\\ y_{num} & =\left\lfloor \frac{y_{max}-y_{min}}{L}\right\rfloor +1 \end{array}\right.$$

The data points $p_{i}={\left(x_{i},y_{i}\right)}^{T}$ are assigned to the corresponding grid cell (u,v), establishing a mapping relationship between them. Here, $u\in [0,x_{\mathrm{num}}]$ and $v\in [0,y_{\mathrm{num}}]$. Subsequently, the grid cells can be classified into two categories based on the presence of data points: “occupied grids” that contain data points and “empty grids” that lack data points. However, due to the non-uniform distribution of point clouds in the plane, the use of small grid sizes can lead to the occurrence of isolated “empty grids”, in which case the neighbouring grids may erroneously be classified as boundary grids. To address this issue, a filling operation is employed to populate these “empty grids”, thereby mitigating the risk of misidentifying certain data points as boundary points. Bilinear interpolation is commonly used for this purpose.

#### 3.3.2. Boundary Point Cloud Grid Identification

In the grid structure, the general principle for identifying boundary point cloud grids involves assessing the quantity of “empty grids” among the neighboring grids, which is determined by the boundary property. Specifically, for each “occupied grid” within the structure, the number of “empty grids” among its eight adjacent grids is evaluated. A grid is classified as a boundary grid if it has more than one empty neighbor, while grids without such neighbors are not considered as boundaries. The identification of boundary grids facilitates a coarse approximation of the overall shape of the point cloud contours, distinguishing them from “empty grids” that lack boundary characteristics. This principle serves as a basis for identifying boundary grids and extracting pertinent information regarding the boundaries of the point cloud.

#### 3.3.3. Boundary Extraction

**Figure 6:**
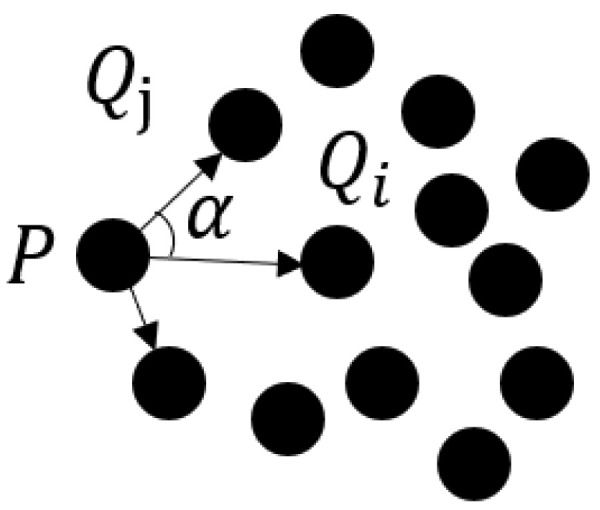
Illustration of point cloud angles.

**Figure 7:**
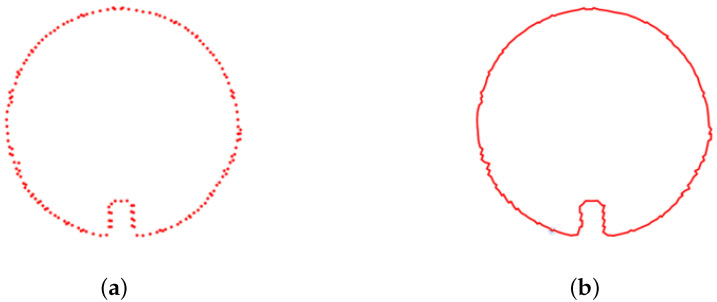
Processed contour diagrams (**a**) Extracted boundary points (**b**) Organized pipeline contour.

The extraction of boundary lines involves refining initially identified boundary point cloud grids, which provide only an approximate shape of the point cloud contours. This falls short of the required accuracy for engineering applications. The subsequent task entails extracting boundary points from each grid and connecting them sequentially to establish the initial boundary lines. Critical to this process is the distribution of neighboring points surrounding boundary points. Typically, these neighboring points exhibit a non-uniform concentration on one side of the boundary, influenced by the contour shape represented by the boundary points. For instance, in pipeline point cloud data, where boundary points correspond to pipe edges, the neighboring points within the interior of the pipes tend to be less concentrated. This non-uniform distribution pattern arises from the inherent geometric shape of the pipes, with a noticeable concentration of neighboring points around the boundary points in the edge region compared with the interior. To address this non-uniform distribution, an algorithm utilizes the standard deviation of angles to measure point cloud distribution uniformity and extract boundary points. The algorithm follows these steps:

1. The k-nearest neighbors of a candidate point and the angles between the candidate point and each neighbor, denoted as *P* and α, are searched and computed respectively. The angle α is defined as shown in Figure 6. Let ${\vec{PQ}}_{i}$ represent the vector between the candidate point *P* and the closest point $Q_{i}$, and let ${\vec{PQ}}_{j}$ represent the vectors between *P* and the remaining points, where j∈(1,k−1). The angles α between ${\vec{PQ}}_{i}$ and ${\vec{PQ}}_{j}$ are calculated to obtain a sequence of angles $\alpha_{i}\left(i=1,2,\ldots ,k-1\right)$.
2. The k-nearest neighbors are sorted in ascending order based on the sequence of angles $\alpha_{i}$ to obtain a sorted sequence of angles $\beta_{i}$.
3. The standard deviations *S* of the sorted sequence of angles are calculated. Points that exceeds a certain threshold value for *S* are identified as boundary feature points. To calculate the angle standard deviation, the angular difference *D* of $\beta_{i}$ is defined as:
  (9) $$D_{i}=\left\{\begin{array}{cc} \beta_{i\;+\;1}-\beta_{i} & \mathrm{if}\;1\leq i<k-1\\ \beta_{i}-\alpha_{1}^{\prime } & \mathrm{if}\;i=k-1 \end{array}\right.$$
  If the standard deviation *S* surpasses the predetermined threshold, the point is categorized as a boundary point due to an uneven distribution of its k-nearest neighbors. Conversely, if *S* is less than or equal to the specified threshold, the point is deemed a non-boundary point. The threshold can be tuned according to the complexity of the point cloud data boundaries, yielding an unordered set of boundary feature points. Figure 7a depicts the boundary points extracted after downsampling. These processed point clouds reveal distinct pipeline contour lines, indicating a regular cylindrical shape of the pipelines under the condition of no deformation or sedimentation. To precisely calculate the cross-sectional area enclosed by the point cloud boundary, interpolation processing is applied to enhance the accuracy of the contour area calculation.
4. To arrange the unordered set of boundary feature points, a k-d tree-based nearest neighbor search algorithm is utilized. This method leverages the k-d tree structure to efficiently search for the nearest neighbors within the point cloud, identifying and sorting the boundary feature points based on their proximity. Connecting the sorted point sequence reveals the boundary lines of the point cloud. Figure 7b depicts the outlined pipeline contours after organizing the boundary points.

Based on the grid-based method described above, the identification and extraction of the outer contour on the projected plane of the pipeline can be achieved. This process lays the foundation for subsequent experimental analysis.

### 3.4. Cross Sectional Area Calculation

**Figure 8:**
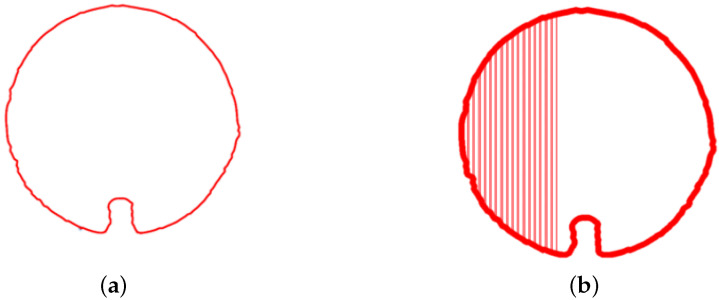
Processed contour diagrams (**a**) Smoothed pipeline contour (**b**) Diagram of cross sectional area calculation.

To further improve the smoothness and continuity of the boundary lines, a smoothing process is applied to the initial boundary, resulting in the final outline of the point cloud. Figure 8a illustrates the smoothed interpolated pipeline contour. To calculate the cross-sectional area of the smoothed pipeline contour lines, integration is performed on the areas enclosed by each point cloud contour line, as depicted in Figure 8b. This allows for a comparison between the calculated area and the known cross-sectional area of the pipeline. Such a comparison facilitates the assessment of pipeline deformation and sedimentation, enabling the detection and analysis of the internal space within the pipeline.

## 4. Experimental Results Analysis

**Figure 9:**
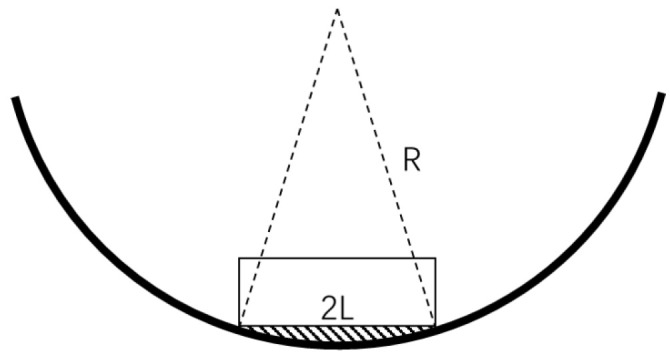
Diagram of unscannable void area.

For the convenient quantification of the level of blockage in this experiment, three representative obstructive materials are employed: stone bricks, paper boxes, and plastic foam, each possessing distinct but regular shapes. In consideration of the unscannable void area formed by the square base and the circular inner wall of the pipe, as illustrated in Figure 9, the experiment involves the calculation of the area of this shaded region. The specific area $S_{v}oid$ is detailed in Equation (10), taking into account the radius *R* of the inner wall of the pipe and the length 2L of the blockage base. Therefore, the whole missing area *S* can be expressed as $S=S_{void}+S_{blockage}$, where $S_{blockage}$ denotes the cross sectional area of the blockage.

(10) $$S_{void}=arcsin\left(\frac{L}{R}\right)*R^{2}-L*\sqrt{R^{2}-L^{2}}$$

Table 1 compares the computed open ratio, derived from the pipeline contour obtained through scanned point cloud data, with the original pipe sectional area of 1824.67 cm${}^{2}$. The comparison between the computed open ratio and the original scenario offers valuable insights into the effectiveness of the proposed method. When influenced by three representative obstructive materials, it is noted that the disparity between the computed open ratio and the actual open ratio is relatively small. This observation underscores the method’s capability to accurately assess the degree of blockage within the pipeline. Thus, the method exhibits high reliability and effectiveness in detecting and analyzing the internal space of the pipeline. It facilitates a quick and precise assessment of pipeline blockage based on point cloud contour data, providing a significant reference for pipeline maintenance and repair.

## 5. Conclusions and Discussion

This study introduces a quantitative method for detecting internal deformations in underground pipelines using laser point cloud data. The proposed approach constructs a 3D scanning system based on laser radar, enabling objective and accurate detection of internal deformations and quantitative assessment of open ratios. Key steps involve determining the pipeline’s direction, clipping point cloud data, plane projection, downsampling, detecting point cloud contours, and transforming and interpolating point cloud data. In contrast to traditional Closed-Circuit Television (CCTV) inspection methods, this approach utilizes high-precision point cloud data from a laser radar’s 3D scanning technology, addressing subjectivity and accuracy limitations in the detection process. It facilitates precise identification of internal pipeline deformations and the calculation of open ratios, providing a scientific foundation for pipeline assessment. The research findings underscore the feasibility and accuracy of the quantitative detection method based on laser point cloud data, offering crucial technical support for maintenance and repair efforts. Future research could enhance practical application by integrating machine learning and image processing techniques to automate the algorithm for complex underground pipeline inspections.

## Figures and Tables

**Table 1:** Comparison of pipeline open ratios for different blockages.

Type	Stone Brick	Paper Box	Plastic Foam
Real view	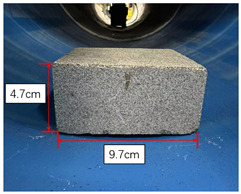	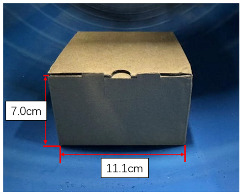	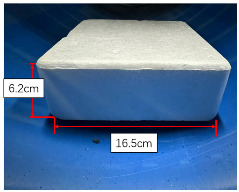
Scanned contour	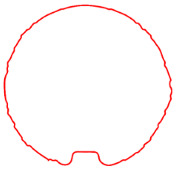	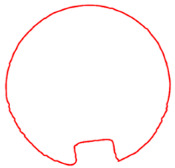	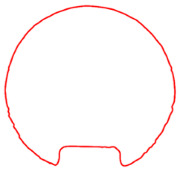
Actual open area (cm${}^{2}$)	1775.88	1742.16	1706.25
Actual open ratio	97.33%	95.48%	93.51%
Calculated open area (cm${}^{2}$)	1771.72	1716.97	1685.70
Calculated open ratio	97.10%	94.10%	92.38%
Measurement error	0.23%	1.38%	1.13%

## Data Availability

The data presented in this study are available on request from the corresponding author.
